# Case Report: Long-term response to multimodal treatment in metastatic uveal melanoma

**DOI:** 10.3389/fimmu.2026.1749363

**Published:** 2026-04-24

**Authors:** Francesca Rifaldi, Martina Angi, Giuseppe Leone, Marta De Ponti, Marta Vaiani, Andrea Spagnoletti, Alice Indini, Michele Del Vecchio, Lorenza Alessia Di Guardo

**Affiliations:** 1 Melanoma Medical Oncology Unit, Department of Medical Oncology ed Hematology, Fondazione Istituto di Ricovero e Cura a Carattere Scientifico (IRCCS) Istituto Nazionale dei Tumori, Milan, Italy; 2 Department of Oncology, Comprehensive Cancer Center, Istituto di Ricovero e Cura a Carattere Scientifico (IRCCS) Policlinico San Matteo Foundation, Pavia, Italy; 3 Department of Internal Medicine and Medical Therapy, University of Pavia, Pavia, Italy; 4 Ocular Oncology Unit, Department of Surgical Oncology, Fondazione Istituto di Ricovero e Cura a Carattere Scientifico (IRCCS) Istituto Nazionale dei Tumori, Milan, Italy; 5 Department of Radiology, Fondazione Istituto di Ricovero e Cura a Carattere Scientifico (IRCCS) Istituto Nazionale dei Tumori, Milan, Italy

**Keywords:** ImmTAC, radiotherapy, synergistic immunologic response, tebentafusp, uveal melanoma

## Abstract

**Background:**

Uveal melanoma (UM) is a rare intraocular malignancy with limited systemic treatment options and poor outcomes once metastatic. Tebentafusp, an ImmTAC (Immune-mobilizing monoclonal T-cell receptor Against Cancer) targeting gp100 in HLA-A*02:01–positive patients, has improved survival, although durable responses remain uncommon. Radiotherapy (RT) has been shown to induce immunogenic cell death and to modulate antitumor immune responses, supporting a potential synergistic interaction with systemic immunomodulatory therapies.

**Case presentation:**

We described a 39-year-old woman with localized UM initially treated with proton therapy (60 Gy in four fractions), who developed liver and hilar adenopathies metastases seven years later. After confirmation of metastatic melanoma and HLA-A*02:01 positivity, tebentafusp was started and well tolerated. Following four months of therapy, imaging revealed oligoprogression in a hilar adenopathy, while hepatic lesions remained stable. Stereotactic body radiotherapy (SBRT; 30 Gy in 10 fractions) was delivered to the nodal site while tebentafusp was continued. Subsequent MRI at nine months demonstrated partial response across all lesions. The patient has maintained disease control for an additional 35 months, with excellent quality of life (ECOG PS 0).

**Conclusion:**

This case illustrates a durable systemic response after combined tebentafusp and SBRT, suggesting a synergistic interaction between ImmTAC-mediated T-cell activation and radiation-induced immunogenic modulation. Local RT may enhance antigen release and T-cell recruitment, amplifying tebentafusp efficacy and inducing abscopal-like effects. Prospective studies are warranted to evaluate this combination and identify biomarkers predictive of response.

## Introduction

Uveal melanoma (UM) arises from melanocytes in the uveal tract of the eye: about 3–5% originate in the iris, 5–8% in the ciliary body, and nearly 90% in the choroid (1). Although it is the most common primary intraocular malignancy in adults, UM is rare overall—roughly 1 case per 100,000 people per year in Europe and 5.3–10.9 cases per million worldwide (2). Major risk factors for UM include fair skin and congenital ocular or oculodermal melanocytosis. The presence of cutaneous, iris, or choroidal nevi may also contribute to risk, while familial and genetic predispositions—particularly germline mutations in BAP1—have been reported. Environmental and occupational exposures, such as arc welding, have been suggested, although the role of ultraviolet radiation remains unclear (3–5).

UM typically exhibits a low tumor mutational burden and recurrent mutations in GNAQ, GNA11, and BAP1, which contribute to its unique biology and the distinct characteristics of its tumor immune microenvironment (TME). The UM TME is generally immunologically “cold,” with limited neoantigen load and a predominance of immunosuppressive cell populations, such as M2-like macrophages and regulatory T cells. Effector T cells are often scarce, and tumor cells exploit immune-evasion mechanisms, including checkpoint molecule expression and suppressive cytokine secretion. These features help explain the limited activity of conventional immune checkpoint inhibitors in UM and provide a rationale for exploring alternative immunotherapeutic approaches (4–6).

The uveal tract is an immunologically privileged site, preserving visual function by limiting local inflammation. This is achieved through immunosuppressive cytokines, regulatory immune cells, and checkpoint molecule expression. Even in healthy eyes, elevated tear cytokine levels reflect baseline inflammation that supports local immune tolerance (7, 8). In UM, this environment reinforces the “cold” tumor microenvironment, limiting T-cell infiltration and influencing both disease progression and responses to immunotherapy.

Despite good local control of the primary tumor, about 50% of patients develop metastases—most commonly to the liver (~90%), followed by the lungs (~24%) and, less frequently, to skin, soft tissue, or bone (~10%) (6). Historically, metastatic UM has been associated with poor median overall survival (OS) of 6–12 months (6). Recent real-world evidence indicates that multimodal management, including tebentafusp and liver-directed therapies, can improve outcomes, with median OS reaching approximately 20–22 months in contemporary cohorts and 21.6 months at 3-year follow-up in the phase 3 tebentafusp trial versus 16.9 months with investigator’s choice (9, 10).

New immunotherapies that redirect cytotoxic T cells toward tumor antigens are promising (11, 12). ImmTACs (Immune-mobilizing monoclonal T-cell receptors Against Cancer) are bispecific fusion proteins that combine engineered, high-affinity T-cell receptors that recognize peptide–HLA complexes (including peptides from intracellular proteins) with an effector domain that engages polyclonal T cells via CD3. This engagement triggers T-cell activation, cytokine release, and tumor-cell lysis (13).

Tebentafusp, the first approved ImmTAC targeting gp100 in the context of HLA-A*02:01, represents a milestone in the treatment of metastatic uveal melanoma. In the pivotal phase III IMCgp100-202 trial, tebentafusp significantly improved median overall survival compared with investigator’s choice therapy, reaching 21.6 months versus 16.9 months at 3-year follow-up. Despite this survival benefit, durable objective responses remain uncommon, and most patients progress within the first year. Importantly, real-world data confirm these findings, showing that patients treated with tebentafusp outside of clinical trials achieve a median overall survival of approximately 21–22 months, supporting the effectiveness of this T-cell redirecting therapy across broader clinical settings (10, 14, 15).

Because the liver is the dominant site of metastasis in uveal melanoma, liver-directed therapies (LDTs) such as radiofrequency ablation, transarterial chemoembolization or immunoembolization and radiotherapy are frequently employed. While these approaches can contribute to disease control and may improve outcomes in selected patients, there are no head-to-head comparisons with systemic therapies, and the optimal selection of LDT is largely dependent on the expertise and protocols of individual treatment centers (11, 16).

Combining stereotactic radiotherapy (SRT/SBRT) with immunotherapy has shown encouraging results in early studies of metastatic uveal melanoma, with retrospective analyses reporting enhanced local control and indications of systemic benefit and acceptable toxicity when added to immune checkpoint inhibition or other systemic therapies (17). In addition to improved local responses, recent work suggests that concurrent or sequential local therapy may extend progression-free intervals and contribute to overall disease control in select patient cohorts, although prospective evidence remains limited and optimal integration strategies are still under investigation (15).

We report the first documented case of a young woman with UM and metachronous liver and nodal metastases who achieved an exceptional response after combined tebentafusp and local SBRT. We propose that a synergistic interaction between ImmTACs and ablative radiotherapy may be possible and warrant prospective evaluation.

## Case report

A 39-old woman with no comorbidities and with no family history of malignancies received a diagnosis of localized uveal melanoma in November 2015.

**Figure 1:**
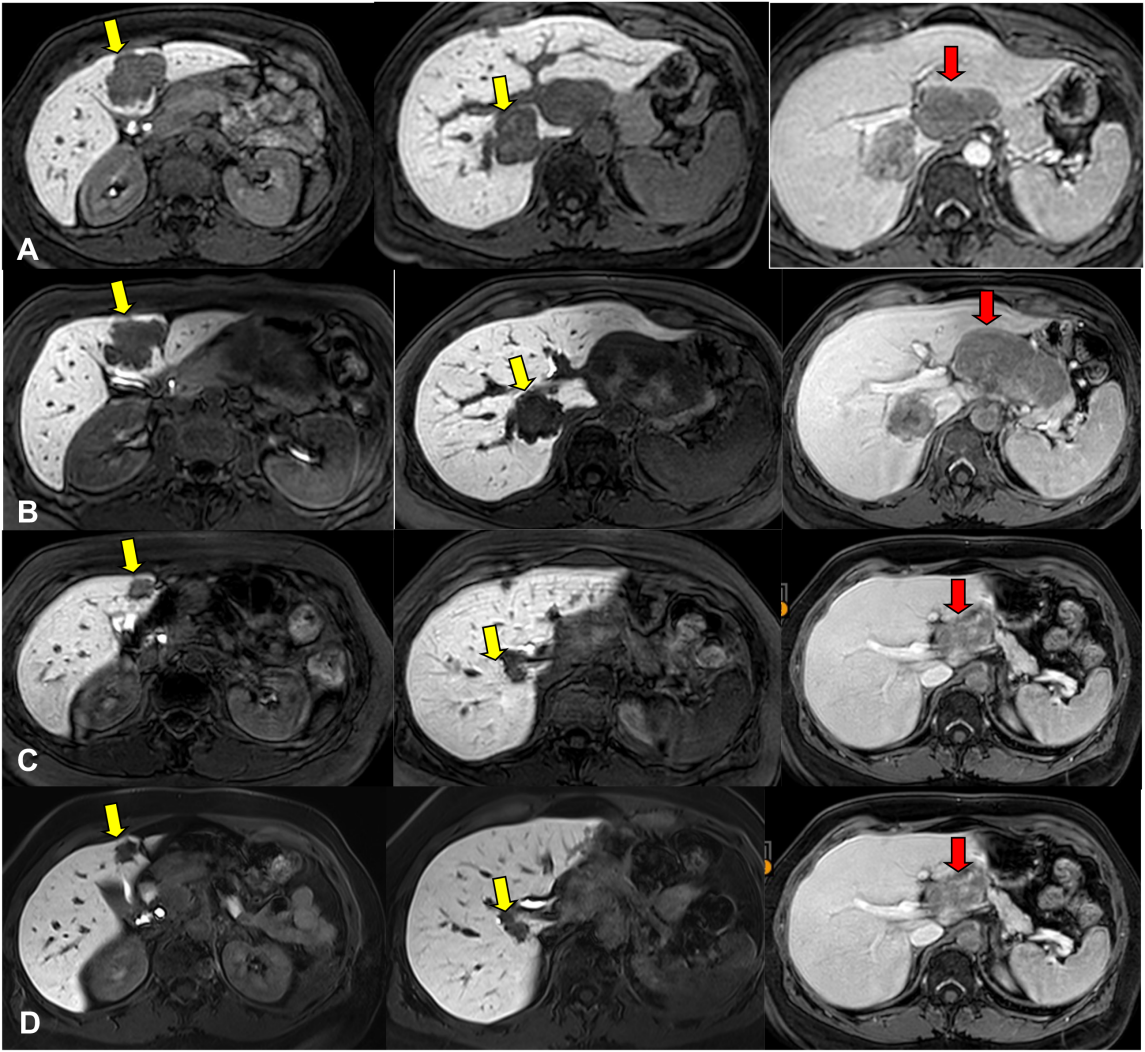
MR images acquired using a 1.5 T scanner (Philips Ingenia) showing: **(A)** liver lesions in segments 4B and 7 **(A, B,** yellow arrows**)** acquired during the hepatobiliary phase. Hilar adenopathy is shown in the venous T1 phase **(C,** red arrow**)**. The images were obtained in November 2022, prior the initiation of systemic therapy. **(B)** liver lesions in segments 4B and 7 **(A, B**, yellow arrows**)** acquired during the hepatobiliary phase. Hilar adenopathy is shown in the venous T1 phase **(C,** red arrow**)**. The images were obtained in April 2023, prior the initiation of radiotherapy. **(C)** liver lesions in segments 4B and 7 (A e B, yellow arrows) acquired during the hepatobiliary phase. Hilar adenopathy is shown in the venous T1 phase **(C,** red arrow**)**. The images were obtained in March 2024, six months after the end of the radiotherapy. **(D)** showing liver lesions in segments 4B and 7 **(A, B,** yellow arrows**)** acquired during the hepatobiliary phase. Hilar adenopathy is shown in the venous T1 phase **(C,** red arrow**)**. The images refer to the last MR evaluation in May 2025.

She underwent proton therapy (60 Gy in 4 fractions) with complete response and negative follow up until September 2022, when multiple liver lesions (six target lesions) and a hilar adenopathy (45x30 mm) appeared, in a completely asymptomatic patient (**Figure 1A**).

Liver biopsy was performed because of the long period (7 years) between first diagnosis and metastatic spread. The histopathology report confirmed localization of melanoma; NGS analysis conducted on the newly obtained biopsy showed the presence of GNAQ mutation.

As the patient was positive for allelic variation HLA-A*02:01, she received systemic treatment with Tebentafusp (Following the tebentafusp dose-escalation schedule, the patient received 20 µg for the first administration, 30 µg for the second, and 68 µg from the third administration onwards) from December 2022. Treatment was well tolerated (rash and fever G1) in the first three cycles.

At the second radiological evaluation there was an increase in the target lesion corresponding to adenopathy in hepatic hilum, while liver lesions were stable (**Figure 1B**).

Given the oligo progression after four months of treatment in a clinically symptomatic patient, presenting with pain due to compressive effects from an enlarging adenopathy, the patient underwent SBRT on adenopathy in the hepatic hilum (30 Gy in 10 fractions) and continued with systemic treatment.

In September 2023 an abdomen MRI was repeated with evidence of partial response in all lesions.

In May 2024 the patient was discussed in a multidisciplinary team, but surgical indication was excluded because of the persistence of multiple liver lesions in both hepatic lobes. Considering the great response to Tebentafusp and excellent tolerability, we decided to continue this treatment (**Figure 1C**).

**Figure 2:**
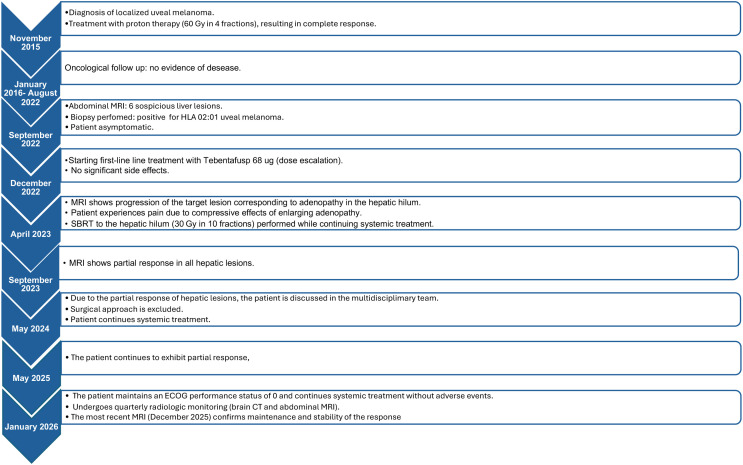
Timeline of diagnosis, treatment, and clinical outcomes of the presented patient.

After three years of therapy (**Figure 1D**), the patient continues to exhibit a partial response and maintains excellent quality of life (ECOG PS 0). She undergoes quarterly radiologic monitoring, including contrast-enhanced CT of the brain and thorax and abdominal MRI, and has remained fully adherent to treatment without any detectable adverse effects, reflecting both the tolerability of therapy and the effectiveness of close surveillance (**Figure 2**).

## Discussion

Uveal melanoma (UM) is a rare malignancy, with approximately 6,500 new cases diagnosed worldwide each year, predominantly among Caucasian patients. Primary management typically involves radiation therapy—brachytherapy or proton beam—or surgical enucleation. Despite achieving effective local control, nearly half of patients will eventually develop metastatic disease, most commonly affecting the liver (~90%), with less frequent spread to the lung, bone, and skin. Risk of relapse peaks between 1 and 5 years after diagnosis and is higher in patients over 50. Larger primary tumor size, higher mitotic rate, and recurrent genomic alterations such as monosomy 3 and 8q amplification are well-established prognostic factors, highlighting the importance of genomic profiling for risk stratification and surveillance planning (3–5).

Systemic options for metastatic UM (mUM) remain limited. Immune checkpoint inhibitors (ICIs) have generally produced only modest and transient benefits. Contributing factors include the tumor’s low mutational burden, low PD-L1 expression, and an immunosuppressive tumor microenvironment evolved to protect ocular tissues (18–20).

Radiotherapy (RT) can complement systemic therapy by inducing immunogenic cell death (ICD) and, in selected contexts, eliciting the so-called “abscopal effect,” wherein regression is observed in distant, non-irradiated lesions. By converting the irradiated lesion into an *in situ* vaccine, focal RT enhances antigen presentation and systemic immune recognition. Nonetheless, true abscopal responses are rare, as metastatic tumors deploy multiple mechanisms to suppress systemic immunity, and RT alone is often insufficient to overcome these barriers (19–21). Mechanistically, RT can activate tumor-specific cytotoxic T cells within the irradiated microenvironment, while ICIs relieve inhibitory checkpoints. Cytokines such as IFN-γ and TNF-α released by reactivated T cells further mediate tumor cell killing, potentially contributing to systemic antitumor responses (22, 23).

In our patient, the combination of stereotactic RT (SBRT) to hilar lymph nodes and systemic tebentafusp produced a notable and durable clinical response. SBRT likely promoted the release of tumor antigens, including neoantigens and DAMPs, while reactivating previously anergic T cells. Tebentafusp, in turn, recruited and activated polyfunctional T cells specifically against gp100–HLA-A*02:01–expressing tumor cells. Unlike conventional ICIs, tebentafusp can redirect polyclonal T cells to tumor targets even within an immunologically “cold” microenvironment. Preclinical and clinical data suggest that the synergy of RT and tebentafusp may involve mechanisms such as HLA-I upregulation and vascular modulation, facilitating T-cell trafficking to non-irradiated metastases and amplifying systemic cytokine-mediated tumor control (16, 17, 24).

The immunologic effects of SBRT are dose-dependent, with ablative regimens particularly effective at inducing ICD and enhancing systemic immune stimulation, potentially contributing to radscopal effects (25). Tebentafusp itself has been shown to activate T cells, increase cytokine production, and promote tumor infiltration, providing a complementary systemic immune stimulus (22).

Several caveats should be considered. Delayed responses to tebentafusp, including partial or complete responses observed on follow-up imaging, have been documented in controlled trials and in real-world reports (23). Furthermore, intrinsic tumor biology plays a role; a long metastasis-free interval—as observed in our patient, seven years—is generally associated with a more favorable prognosis (26). Dissociation between RECIST-defined response and clinical benefit with tebentafusp has also been described, emphasizing that radiographic progression does not always reflect lack of therapeutic effect.

Ongoing clinical trials are exploring the combination of tebentafusp with liver-directed therapies (LDTs), such as SBRT or transarterial interventions, reflecting a growing interest in multimodal approaches to optimize outcomes (27, 28). Although evidence is still limited and largely anecdotal, this case suggests that integrating focal liver-directed RT with tebentafusp may enhance systemic immune activation and extend time-on-treatment beyond what is achieved with tebentafusp alone.

In conclusion, while abscopal responses in mUM remain rare, our observations support a biologically plausible synergy between SBRT and tebentafusp, mediated by antigen release, polyclonal T-cell recruitment, and systemic cytokine activity. Further translational and prospective studies are needed to fully understand these mechanisms and to define the optimal sequencing and combination strategies in this challenging disease (18–28).

## Conclusion

Since its initial characterization in the 1950s, the abscopal effect has been documented across various tumor types and clinical contexts. Historically, this phenomenon was infrequently reported and predominantly linked to radiation therapy alone. However, the emergence and widespread adoption of novel therapeutic modalities have broadened the understanding and clinical relevance of the abscopal effect, enhancing its potential therapeutic benefit.

To our knowledge, this is the first case report to demonstrate a long-lasting response resulting from the synergistic interaction between local radiotherapy and Tebentafusp, a next-generation immune T cell engager (ImmTAC). Although the findings are encouraging, they are inherently limited by the uniqueness of the case and the relatively short follow-up period. This underscores the need for further studies on combinatorial approaches, as well as the development of personalized combination strategies tailored to individual patients.

Moreover, the identification of various biomarkers may enhance our ability to predict which patients are more likely to experience the abscopal effect.

## Patient perspective

Receiving the diagnosis of metastatic uveal melanoma at 39 was overwhelming, especially as a mother of a teenage son. I was aware of the seriousness and rarity of my condition, and at times I feared I would not have enough time with my family. Despite these worries, the weekly treatment has been surprisingly manageable. I have always felt cared for, supported, understood, and listened to by my medical team, which has made an enormous difference in facing this disease. Seeing such an exceptional response to therapy has given me renewed strength and motivation to continue this journey. For the first time since my diagnosis, I feel hopeful about the future.

## Data Availability

The original contributions presented in the study are included in the article/supplementary material. Further inquiries can be directed to the corresponding author.
